# Exposure versus Susceptibility as Alternative Bases for New Approaches to Surveillance for *Schistosoma japonicum* in Low Transmission Environments

**DOI:** 10.1371/journal.pntd.0004425

**Published:** 2016-03-04

**Authors:** Shuo Wang, Robert C. Spear

**Affiliations:** Center for Occupational and Environmental Health, School of Public Health, University of California, Berkeley, Berkeley, California, United States of America; University of Edinburgh, UNITED KINGDOM

## Abstract

Currently, schistosomiasis in China provides an excellent example of many of the challenges of moving from low transmission to the elimination of transmission for infectious diseases generally. In response to the surveillance dimension of these challenges, we here explore two strategic approaches to inform priorities for the development of improved methods addressed specifically to schistosomiasis in the low transmission environment. We utilize an individually-based model and the exposure data used earlier to explore surveillance strategies, one focused on exposure assessment and the second on our estimates of variability in individual susceptibility in the practical context of the current situation in China and the theoretical context of the behavior of transmission dynamics near the zero state. Our findings suggest that individual susceptibility is the major single determinant of infection intensity in both the low and medium risk environments. We conclude that there is considerable motivation to search for a biomarker of susceptibility to infection in humans, but that there would also be value in a method for monitoring surface waters for the free-swimming forms of the parasite in endemic or formerly endemic environments as an early warning of infection risk.

## Author Summary

Surveillance programs for environmentally-mediated infectious diseases in the developing world present challenges to public health authorities in acquiring adequate resources for their widespread use as well as in the availability of practical and reliable methods. These challenges are exacerbated in cases where transmission is suppressed to low but persistent levels, as is currently the case for the parasitic disease schistosomiasis in China. In this paper we use epidemiologically-based computer modeling to explore the potential utility of new approaches based on human susceptibility on one hand and exposure-related methods on the other. Our intent is to motivate the development of new methods based on one or both of these strategic approaches and aimed at the low transmission environment. We conclude that there is considerable motivation to search for a biomarker of susceptibility to infection in humans and also to develop a method for monitoring surface waters for the free-swimming forms of the parasite in endemic or formerly endemic environments as an early warning of infection risk.

## Introduction

In an earlier paper we reported on the deficiencies of current methods used for surveillance of schistosomiasis transmission in China as both the prevalence and intensity of infection decline in response to continued control efforts [1]. For the most part, these methods are based on case reports or episodic infection surveys focused on human and bovine populations and on the presence of infected *Oncomelania* snails, the intermediate hosts of *Schistosoma japonicum*. It is clear that these methods that have served well in the past are not adequate for low transmission environments when elimination of disease transmission is the ultimate goal. This is due to the fact that low transmission environments are generally characterized by prevalence and intensity of infection in the human population that, in our work, are typically well below 10% prevalence and 10 EPG, eggs per gram of feces, as a village average. In addition, low levels of infection are found in bovines, where they are significant mammalian hosts and few, if any, infected snails are found in these environments using standard survey techniques. Currently, the transmission of *S*. *japonicum* in China provides an excellent example of many of the challenges of moving from low transmission to elimination described by Klepac et al [2] for infectious diseases generally. In response to the surveillance dimension of these challenges, we here explore two strategic approaches to inform priorities for the development of improved methods addressed specifically to the low transmission environment.

A further characteristic of schistosomiasis at low levels of transmission is slow transmission dynamics near the zero state, that is, the disease-free state [3]. In common with lymphatic filariasis and other parasitic diseases, system dynamics are influenced by an unstable equilibrium point between the endemic level of transmission and the zero state, often termed the breakpoint[4]. In the case of schistosomiasis, the breakpoint is quite near the disease-free state and, in mathematical models at least, this results in low but very slowly changing infection levels over periods of years even where the basic reproductive number is less than unity indicating that transmission will eventually cease [5]. Analyses of re-emergent schistosomiasis in China are consistent with this picture of either periods of undetected transmission, that can be on the order of 8–10 years, or reintroduction of the parasite into formerly endemic areas. Yet a further complication is that models suggest that quite small schistosome egg inputs into formerly endemic areas are sufficient to re-establish very low levels of persistent transmission [3]. Hence, even a very few infected humans, bovines, or other mammalian hosts may be barriers to reaching elimination furthering the case for new approaches to surveillance.

Schistosomiasis is widespread in tropical and subtropical areas of the world. The World Health Organization estimates that at least 261 million people received preventive treatment for schistosomiasis in 2013 [6]. For almost two decades our group has focused on the understanding the exposure of rural villagers in areas of Sichuan Province of southwestern China that are historically endemic for transmission of *S*. *japonicum* [7, 8]. In our environment, infection is not strongly influenced by age or sex among school age children or adults [7]. Hence, our objective has been to identify environmental targets for sustainable interventions to suppress transmission in the variety of exposure circumstances inherent in irrigated agricultural settings.

Over this period we have accumulated a database sufficient to support estimation of the annual cercarial exposure of two populations over one to two year intervals, one at medium levels of infection risk common in this region in the late 1990s and early 2000s and a second at the low levels more typical of the post- SARS era which saw broad scale interventions in China aimed at various infectious diseases including *S*. *japonicum*. This database has allowed us to estimate the roles of environmental exposure versus individual susceptibility to infection as determinants of transmission and as targets for surveillance. In particular, we recently reported a statistical analysis of re-infection data in two cohorts which resulted in the finding that individual susceptibility was an important determinant of infection and infection intensity [9]. This finding motivated a more detailed mechanistic analysis of the same data, but using an individually-based mathematical model that incorporated supplemental exposure-relevant environmental data [10]. This analysis suggested that individual variability in susceptibility to infection ranges over at least two orders of magnitude and that those in the upper tails of that distribution, the super-susceptibles, may be responsible for much of the over-dispersed nature of the distribution of worm burden characteristic of helminth infections independently from other heterogeneities in contact rates. Here we utilize the individually-based model and the exposure data used earlier to explore surveillance strategies, one focused on exposure assessment strategies and the second on our estimates of variability in susceptibility in the practical context of the current situation in China and the theoretical context of the behavior of transmission dynamics near the zero state.

## Methods

Both our earlier statistical and mechanistic-based analyses of exposure and susceptibility as determinants of disease transmission can be summarized by the expression *w*_*T*_ = *αC*_*T*_ where *C*_*T*_ is the cumulative number of cercarial hits to an individual’s exposed skin over time interval *T*, *α* the fraction of those hits that penetrate the skin, and *w*_*T*_ the resulting worm burden which incorporates a small stochastic correction for worms that die before reaching maturity *in vivo*. In humans, *w*_*T*_ is generally assumed proportional to schistosome egg excretion in fecal samples, typically quantified using the Kato-Katz procedure and expressed as EPG. Based on estimates of the daily fecal egg concentration per worm pair [11], we assumed that the cumulative number of eggs produced per infected person over the exposure interval *T* is *E*_*T*_ ≈ *αC*_*T*_.

The parameter *α* is the measure of susceptibility used here to represent the result of a complicated chain of biological processes involving the host, the parasite, and their interaction. In a given environment, we assume that any differences in *α* resulting from differences in infectivity within the cercarial population are averaged out over an individual’s exposure time *T* which is typically 1 or 2 years in our data. Hence, we here regard the population variability in *α* as a property of the individual host which we assume to be a stable property over the duration of exposure. At the low levels of infection risk which are the focus here, an earlier analysis suggests that infection acquired immunity is unlikely to be a significant factor, another feature of infectious diseases often observed in moving from low transmission to elimination [2, 12]. Hence, acquired immunity was not included in the model or the analyses reported herein.

The rationale and details of the individually-based model (IBM) central to our analysis, as well as a description of the epidemiological data underlying its parameterization, are given elsewhere but summarized here [10]. The epidemiological data upon which the model is based were collected from two cohorts. The first was comprised of about 1800 people from 10 endemic villages who were tested for infection in 2000, 2002 and 2006. The prevalence at baseline in 2000 was 46.9% and the average EPG was 46. The second is comprised of 1600 people from 53 villages in two counties where schistosomiasis infection had re-emerged after having achieved control status, a criterion for which is prevalence below 1%. In these villages, infection surveys were conducted in 2007, 2008 and 2010 and prevalence at baseline in 2007 was 2.6%. Subsets of individuals for whom longitudinal infection data and extensive water contact data were available and used in the detailed analyses of susceptibility as described previously. They totaled 529 people from the 10 villages subsequently referred to as Cohort 1 and 727 from 28 villages comprising Cohort 2. The simulation studies reported here were of two hypothetical populations, HP1 closely based on the epidemiological characteristics of Cohort 1 and HP2 on those of Cohort 2 [10].

The IBM tracks worm development in each individual and includes water contact, cercarial density in water to which the individual is exposed, individual susceptibility to worm development, and the fraction of parasites surviving to maturity *in vivo*. It is a significant challenge in the low transmission environment to complete the loop from worm burden in a village population through fecal egg excretion, miracidial hatching and transport to cercarial density produced by infected snail populations. First, identifying the spatial and temporal distributions of infected snails and the free-swimming forms of the schistosome within villages using snail surveys and cercarial bioassays central to model calibration in our earlier work, are no longer sufficiently sensitive in the low transmission environment. Further, virtually all of our earlier modeling work was focused on internal transmission among humans and snails within a village in contrast with the very ill-defined external sources of the free-swimming forms of the parasite that are likely to be a much more important contributors to village level infection risk in the low transmission environment [5]. As a result, cercarial density was treated as an input variable to the IBM in order to utilize exposure-related field data available to us from the low prevalence end of our earlier studies and focus on the effects of individual exposure modifiers, together with susceptibility and diagnostic sensitivity, as they condition host response to environments so defined.

Exposure, or the cumulative number of cercarial hits to the exposed skin of the i-th individual over time interval *T*, is estimated by:

$$C_{i}=\sum_{j=1}^{n_{i}}r_{s}s_{i}c_{jk}\Delta t=r_{s}s_{i}\Delta t\sum_{j=1}^{n_{i}}c_{jk}$$

where *T* is divided into *n*_*j*_ exposure episodes of duration *Δt*, and *s*_*i*_ the annual average wetted skin surface in m^2^ per episode. Although the total annual water exposure was determined from survey data, the duration of an exposure episode was fixed at 30 minutes somewhat arbitrarily to allow the simulation of short-term stochastic variations in cercarial density. As mentioned above, the simulated cercarial density during each episode, *c*_*jk*_, is a stochastic variable with a fixed mean value for each village. The assumption of a fixed village mean is based two earlier findings. First, we found little change in average village cercarial density over the infection season [13]. Second, it has been a consistent epidemiological finding over several decades that village of residence is the dominant predictor of individual infection intensity with occupation being the second most important factor [11, 14]. In the foregoing equation, the effect of occupation is reflected in the parameter *s*_*i*_. The parameter *r*_*s*_ was initially included as a modifier of the cercarial concentration in water to the fraction of those that attached to exposed skin. However, it was found that the field data used to estimate the cercarial density was, in fact, an estimate of *r*_*s*_*c*_*j*,*k*_. Hence *r*_*s*_ was set to unity, but included for unitary consistency in these analyses.

Based on limited infection data, earlier we used the model to estimate the susceptibility parameter *α*, the probability that a cercariae that penetrates the intact skin develops into an adult worm [10]. Specifically,

$$\alpha_{i}\approx \frac{E_{i}}{r_{s}s_{i}\Delta t}\cdot \frac{1}{\sum_{j=1}^{n_{i}}c_{j}}$$

where *E*_*i*_ is the cumulative egg excretion of the i-th individual. This estimate was based on complete data from 132 individuals from our two cohorts that constituted the upper 12 percentile of the *α* distribution for the combined population. There was no compelling evidence that the *α* estimates from the two infected populations differed hence, a single distribution was assumed to represent both. A match to the key summary statistics of infection prevalence and intensity data for the two populations separately was used to extrapolate the lower part of *α* distribution. The best match was obtained with a log-normal distribution with a geometric standard deviation of 4.5 and an 88^th^ percentile value matching the low end of the re-infection estimates. This distribution was used in the simulation studies reported here. Clearly, there is a strong speculative element to this choice and it may be subject to the effects of various confounding factors. This possibility will be seen to underscore our argument that new methods for estimating *α* much more directly would be very valuable.

Other key features of the model are based on the assumptions that any male and female worms that reach maturity in the host pair with probability one but that the assigned sex of each adult worm reaching maturity is random. In addition, estimates are assumed regarding the sensitivity of the Kato-Katz procedure and the miracidial hatch test used in China to detect infections in humans [15].

As noted above, the simulation is of the exposure and subsequent worm development of 529 people from the 10 villages of HP1 and 727 people from 28 villages comprising HP2. The epidemiological data underlying these hypothetical populations was gathered after a 2 year period of exposure for HP1 and a 1 year exposure for HP2. In both cases, the exposure period began after a diagnostic survey in which all infected individuals were treated with praziquantel. In the simulations we assume treatment effectiveness to be 100% which results in all initial worm burdens being set to zero.

We now address the potential utility of population surveillance based on individual exposure estimates versus surveillance based on measures of susceptibility. In neither case do methods currently exist to match what we can simply record from model simulations. Hence, the objective is to use the model to suggest research priorities for surveillance methods that might be cheaper and more effective than conducting episodic infection surveys or follow-up investigations of case reports of acute infections. The most obvious examples of potentially useful tools would be a method for measurement of schistosome cercariae in surface water on one hand or the identification of a human biomarker of susceptibility on the other. The issue is to determine their relative effectiveness if such methods were available.

### Ethics Statement

Although all individual identifying data had been removed prior to the analyses reported herein, the original data collection and management was approved by the Berkeley IRB (Protocol 2010-02-877) and the Sichuan IRB (Protocol 2008–03).

## Results

**Fig 1:**
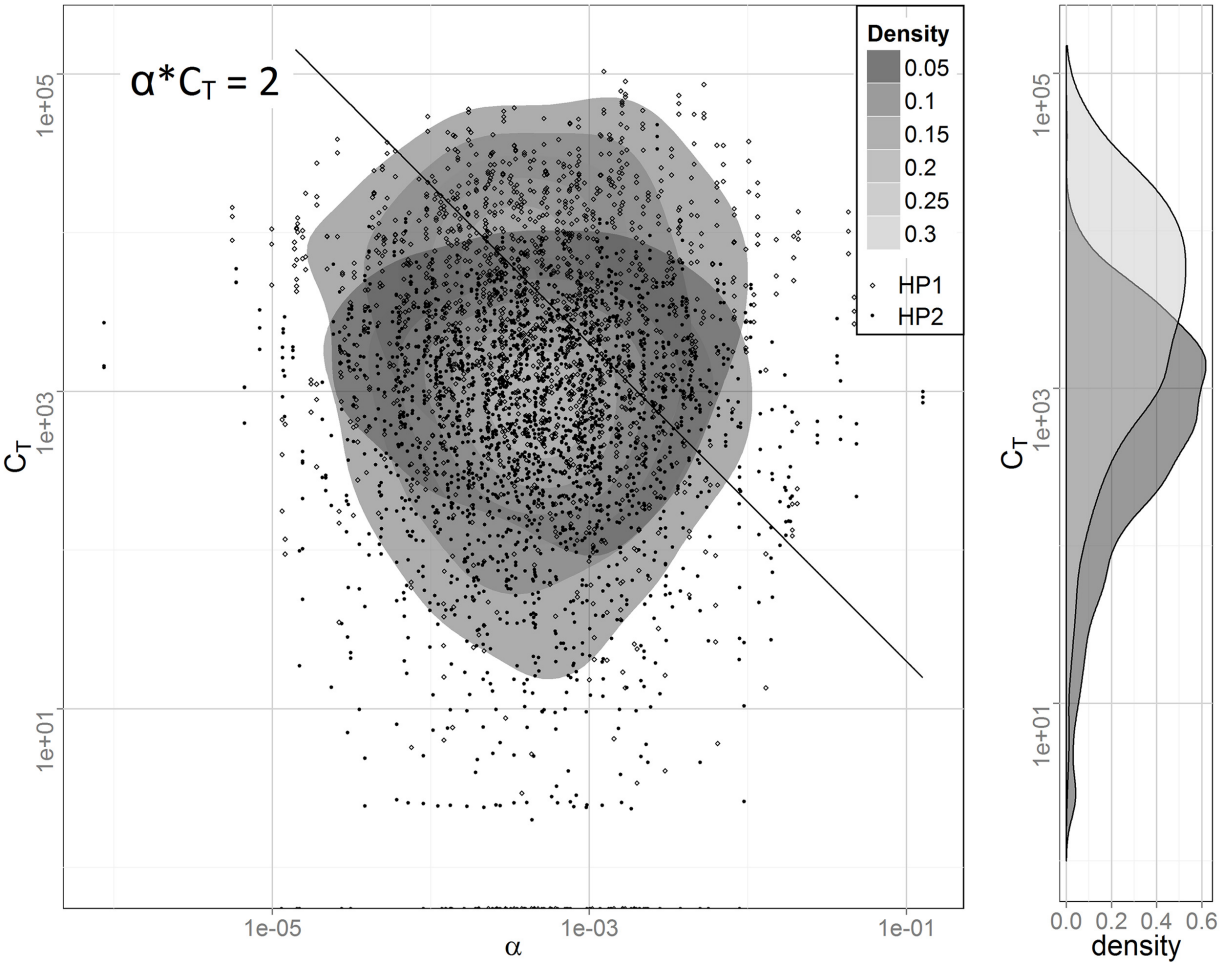
The bivariate distribution of the susceptibility parameter *α* and cumulative cercarial exposure *C*_*T*_ together with the marginal distribution of *C*_*T*_ for simulated populations HP1 (light) and HP2 (dark). In both populations the distribution of *α* is log normal with a geometric standard deviation of 4.5. *αC*_*T*_ = 2 is the lower bound on the number of worms necessary to produce schistosome eggs.

**Fig 2:**
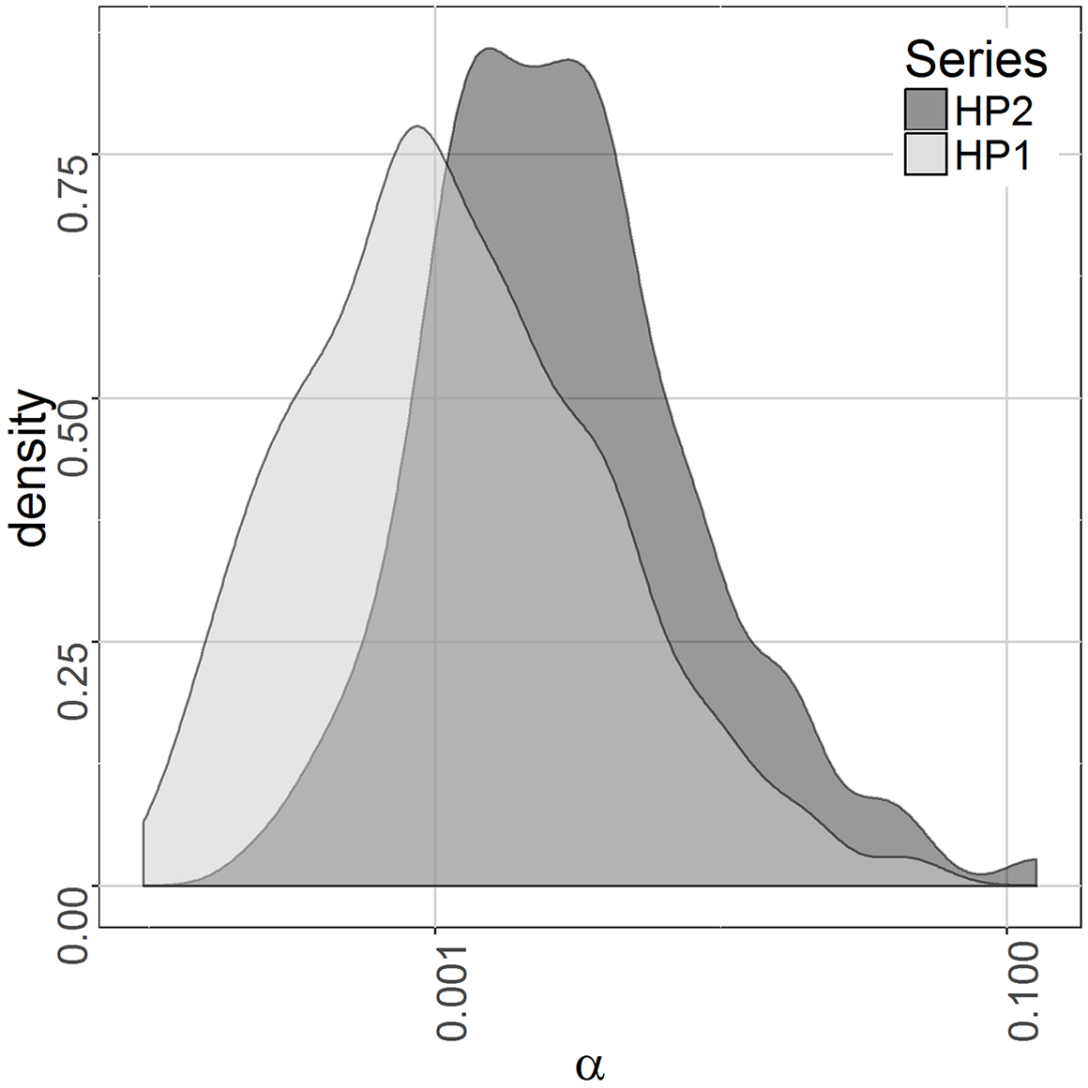
Observed distributions of the susceptibility parameter *α* for those infected in simulated populations HP1 and HP2.

Here we first confirm that the qualitative characteristics we speculated to underlie our earlier statistical results of the two cohorts also underlie the simulation results. In particular, we speculated that the increased ratio of observed to expected reinfections in the less exposed population, as compared to the more exposed population, was attributable to the fact that only a more highly susceptible fraction of that population was at risk of reinfection [9]. The situation is illustrated in Fig 1 which shows a log-log plot of the bivariate distribution of *α* and *C*_*T*_ and the marginal distribution of total cercarial hits, *C*_*T*_. The kernel density estimation procedure stat_density2d in ggplot2 from the statistical package R was used. The diagonal line in the figure corresponds to *w = 2* since that is the lower bound on the number of worms necessary to produce schistosome eggs. That is, all infected individuals lie above and to the right of the line together with a few uninfected due to two worms of the same sex or because their light infections were not identified by the diagnostic tests used which can miss 25% to 50% of infections at mean EPG values less than 10 [15]. The differences in exposure, *C*_*T*,_ are due both to higher cercarial densities associated with HP1 and the two- versus one-year exposure period. Fig 2 shows the distributions of the susceptibility parameter *α* for those infected in the two populations. (Note that the *α* axis is logarithmic.) As can be seen, infected individuals from the population with the lower exposure, HP2, show a higher average susceptibility than those from HP1. This supports the contention that individuals from the right tail of the susceptibility distribution play an increasingly important role in sustaining transmission as exposure intensity declines. Here we pursue the implications of this observation, using the model, in the context of disease surveillance.

The individual characteristics available from the simulation for each exposure interval *T* are the susceptibility parameter *α*, the number of exposure events experienced by each, *n*_*i*_, their time-weighted average water contact per exposure event, *s*_*i*_, and the mean cercarial density experienced by each individual during these exposures, ${\bar{c}}_{v}$. If it were possible to estimate each of these parameters combinations *a priori* for the entire population, Tables 1 and 2 show the percent of infected populations HP1 and HP2, respectively, and the percent of total population egg excretion of these individuals deposited in the environment for various percentiles of those tracked. For example, for simulated population HP1, if the number of 30-minute water exposure episodes, *n*_*i*_, were known for all individuals, those who experienced the top 10% of all exposure episodes would have accounted for 32.6% of the total egg excretion and 19.8% of those infected. If knowledge of individual water contact per exposure, *s*_*i*_, is added, these fractions increase to 38.6% and 21.2% respectively, with a further increase to 49.4% and 24.8% if cercarial density, ${\bar{c}}_{v}$ is added. The combination $n_{i}s_{i}{\bar{c}}_{v}$ indicates that complete exposure information is known for each person and, for example, if one focused on treating the most exposed 10% of HP1, half of the egg contamination that these individuals would have contributed to the village environment would be prevented.

In contrast, upper percentiles of the susceptibility parameter, *α*_*t*_, account for remarkably large percentiles of egg excretion, but lesser percentiles of infection. The top 10% of susceptibles in HP1, for example, account for 62.3% of egg excretion and 25.4% of infections. The top 20% of susceptibles account for 78.8% and 82.3% of egg excretion in the two cohorts. In all cases for both HP1 and HP2, the susceptible percentile accounts for higher egg excretion fractions and proportions of infections than does the corresponding percentile of any exposure-related metric.

**Table 1:** Percentiles of the population HP1 detected as infected or the percentiles of total population egg excretion, EPG, accounted for by upper percentiles of the distributions of susceptibility, *α*_*i*_, number of exposure episodes, *n*_*i*_, cumulative water contact, *n*_*i*_*s*_*i*_, and cumulative cercarial exposure, $n_{i}s_{i}{\bar{c}}_{v}$.

Top % of people tracked	Percentage detected (HP1)							
	EPG				Infected (yes/no)			
	*α*_*i*_	*n*_*i*_	*n*_*i*_*s*_*i*_	$n_{i}s_{i}{\bar{c}}_{v}$	*α*_*i*_	*n*_*i*_	*n*_*i*_*s*_*i*_	$n_{i}s_{i}{\bar{c}}_{v}$
5%	47.6	20.1	24.8	33.8	14.2	10.9	11.8	13.8
10%	62.3	32.6	38.6	49.4	25.4	19.8	21.2	24.8
15%	72.1	43.4	49.2	60.7	35.8	28.4	30.0	34.9
20%	78.8	52.1	57.7	68.8	44.8	36.0	37.8	43.7
50%	96.0	85.1	87.7	93.0	82.4	73.3	75.0	81.3

**Table 2:** Percentiles of the population HP2 detected as infected or the percentiles of total population egg excretion, EPG, accounted for by upper percentiles of the distributions of susceptibility, *α*_*i*_, number of exposure episodes, *n*_*i*_, cumulative water contact, *n*_*i*_*s*_*i*_, and cumulative cercarial exposure, $n_{i}s_{i}{\bar{c}}_{v}$.

Top % of people tracked	Percentage detected (HP2)							
	EPG				Infected (yes/no)			
	*α*_*i*_	*n*_*i*_	*n*_*i*_*s*_*i*_	$n_{i}s_{i}{\bar{c}}_{v}$	*α*_*i*_	*n*_*i*_	*n*_*i*_*s*_*i*_	$n_{i}s_{i}{\bar{c}}_{v}$
5%	49.9	24.4	27.3	31.1	28.5	18.2	19.4	21.7
10%	65.4	39.1	41.8	46.4	46.9	31.1	32.7	36.1
15%	75.5	50.5	53.1	57.9	61.1	42.2	44.0	48.0
20%	82.3	59.4	62.0	66.6	71.6	51.6	53.3	57.7
50%	98.4	90.1	91.0	93.2	97.5	86.9	87.7	90.3

## Discussion

A notable aspect of the simulation results summarized in the tables is that, in both HP1 and HP2, those individuals comprising the top 20% of the distributions of susceptibility, *α*_*i*_, account for very close to 80% of the parasite egg excretion to the environment. This is an example of the 20/80 rule for infectious agents generally. This rule was formalized by Woolhouse et al. who argued that for many diseases 20% of the host population contributes at least 80% of the net transmission potential [16]. In their analysis, the 20/80 rule refers to stratification of the population by ‘contact rate’ which includes both susceptibility and exposure as defined here. The importance of the 20/80 rule as outlined by Woolhouse et al is that control programs targeted at the 20% are potentially “highly effective.”

The infected subpopulations of HP1 or HP2 are the sources of parasite eggs excreted into their own environment or that of an extended population of potentially exposed snails and, in turn, other humans. We regard EPG as a measure of host-related net transmission potential since cessation of EPG input to the environment will terminate transmission over time barring external miracidial inputs. The EPG columns of Tables 1 and 2 show the fraction of total egg input to the environment by the infected subpopulations stratified by the various exposure-related measures and by susceptibility. Again, exposure we defined as the number of cercariae impacting an individual’s skin in the exposure interval *T*. As argued above, the issue is to identify these people by means other than large-scale infection surveys.

The most complete of the exposure metrics, that requiring water contract estimates plus an estimate of mean cercarial density in the environment, falls well below the 20/80 rule, but the top 20% of those exposed by this measure still account for about two-thirds of the EPG excretion to the environment. However, since the simulation treats all residents of a village as being exposed to the same average cercarial density over the exposure period, the gain by adding cercarial density to stratification by intensity of water contact alone relates mainly to differences between villages. Within a village, we expect exposure stratification by water contact intensity alone to be equivalent except for stochastic variations which may be non-trivial in the low transmission environment. If a cost-effective means of measuring cercarial and miracidial density in water were available, we speculate that its value would be more as an early warning indicator of the presence of infected mammals or of infected snails than a useful tool for estimating the level of transmission risk.

If, in fact, susceptibility to infection as implemented in our model is at least a slowly varying characteristic of an individual, it would be a very attractive to seek a biomarker of this characteristic as a means of identifying the sub-population of particularly susceptible individuals for ongoing surveillance. While this sub-population is important at medium and high levels of transmission, it is particularly important in low transmission circumstances as illustrated by the striking upward shift in the *α* values among the lower risk population, HP2, in Fig 2.

The semi-stable characteristics of transmission at low levels of transmission and the apparent sensitivity of the process to very modest levels of parasite re-introduction underscore the desirability of new and more sensitive approaches to surveillance [3]. In addition, highly targeted use of praziquantel to treat humans or bovines and niclosamide to control snail populations minimizes the risk of parasite or snail adaption in the long-term. We conclude that there is considerable motivation to search for a biomarker of susceptibility to infection in humans and also to develop a method for monitoring surface waters for the free-swimming forms of the parasite in endemic or formerly endemic environments as an early warning of infection risk.
